# Enabling awareness, agency and participation: Haematology patient experiences of an (in)patient portal's information affordances

**DOI:** 10.1177/20552076241288371

**Published:** 2024-10-18

**Authors:** Simone Schmidt, Adam Boulton, Benita Butler, Timothy Fazio

**Affiliations:** 1 EMR Team, 90134 The Royal Melbourne Hospital, Parkville, VIC, Australia; 2 School of Computing and Information Systems, The University of Melbourne, Carlton, VIC, Australia

**Keywords:** Inpatient portals, patient portals, person-centred care, qualitative research, digital health, patient experience, patient–clinician relationship, sharing information, reflexive thematic analysis, information affordances

## Abstract

This study examines patient experiences of an inpatient portal's information affordances, including access to results, notes, vital signs, medication information and a schedule. Patient participants were recruited from an inpatient ward primarily catering to patients with haematological malignancies including leukaemia and bone marrow transplant recipients at the Royal Melbourne Hospital. Although focused on the inpatient experience of a portal's information affordances accessed via a hospital-provided tablet, due to limited patient access to notes in the inpatient context, this study also explored patient experiences of their outpatient portal notes accessed via their smartphone. This study demonstrates the value of an (in)patient portal in enabling patient awareness, agency and participation in their care. It shows how an (in)patient portal not only helps patients make informed decisions in their care, but can also initiate patient conversation and collaboration with clinicians. This study points how the digital mediation of healthcare can provide greater transparency in the patient–clinician relationship and address the information asymmetry that typically characterises this relationship, particularly in the Australian context, where inpatient portals are still in their infancy.

## Background

Providing patients access to aspects of their record whilst they are admitted in hospital via a portal that extends aspects of the EMR is part of the movement towards increasing transparency in healthcare and supporting person-centred care. Studies on inpatient portals over the last decade, largely from the USA, report patients value access to their healthcare information whilst admitted in hospital.^1–4^ This qualitative study concentrates on the Australian context where patient portals are still in their infancy. Indeed, to our knowledge, this is the first qualitative study in the Australian context that examines patient experiences of an inpatient portal and as a contextualist study contributes to the extant literature in this respect.

The term *affordance* coined by James Gibson in 1977 can be defined as ‘an action possibility formed by the relationship between an agent and its environment’.^5^ In the context of this study, we use the term *information affordance* to consider how the various types of information accessible via an (in)patient portal enable the patient's agency and participation in their healthcare. This study that includes unique themes dedicated to each information affordance departs from many previous studies that speak to multiple information affordances in relation to more general themes.^2,3,6^ The term information affordance is more commonly referred to in studies on patient portals as *functionality*. Significantly, it has been elsewhere noted that there is a dearth of exploration of the value of individual portal functionalities.^7,8^ To address this imbalance, our analysis is structured according to the experienced value and impact of each information affordance. It also functions as a clear guide for clinicians, providing practical and ethical justification for each information affordance. By detailing the benefits of each affordance and, where relevant, how it could be improved from the patient perspective, our study makes a distinct contribution, particularly to contexts like Australia that have less experience in inpatient portal implementation and evaluation such as the UK.^6^ It has also been noted that few studies consider how portals can be improved from the patient perspective, and this factor has been correlated with problems in portal adoption;^9^ our study helps address this lacuna.

The research question that drove our study is: *What is the value and impact of the information affordances of an (in)patient portal from the patient perspective?* Our study examines patient experiences of Epic's MyChart Bedside inpatient portal in a ward primarily catering to patients with haematological malignancies including leukaemia and bone marrow transplant recipients (hereafter referred to as the haematology ward) at The Royal Melbourne Hospital (RMH), an adult, quaternary, teaching hospital. The inpatient portal was *pioneered* in this ward as well as two other contexts in RMH from November 2022. Currently, the inpatient portal is only implemented in these three *pioneer* contexts in RMH.

Throughout this paper where relevant we employ the term *(in)patient* because although our study focuses on patient access to an *inpatient* portal's information affordances whilst admitted in hospital, we also include patient experience of access to their notes via the *outpatient* portal once they are discharged, due to the restricted access to notes in the inpatient context. Our participants only had access to their admission notes in the inpatient context; in the outpatient context they had access to their discharge and consultation notes. In the case of a patient with leukaemia who spends approximately 4–6 weeks in hospital, has a few weeks at home during which they have regular consultations, and then returns to hospital for another 4–6 weeks, the inpatient and outpatient environments form an ongoing treatment cycle. So, to understand as much as we could about our participants’ experiences of notes, we position the outpatient portal as a logical continuation of the inpatient portal. In connecting the inpatient and outpatient portal in this way, this study presents a novel focus that stimulates consideration as to whether there should be a more seamless integration of information affordances between the two contexts, particularly as concerns access to notes.

## Methods

### (In)patient portal information affordances

The MyChart Bedside inpatient portal is accessible via a patient's smartphone or via a hospital provided tablet routinely available to inpatients in the haematology ward. In this study we focused on participant experience of the tablet as this device enables the additional affordances of access to vital signs, and the mapping of both vital signs and results over time and we were interested in understanding our participants’ experiences of these additional affordances. The (in)patient portal's information affordances examined in this study include access to results; notes; vital signs; medication information; as well as a schedule that enables the inpatient to see whether they have any upcoming medical events such as scans. Once discharged from hospital our participants accessed their outpatient notes via the outpatient portal on their smartphone.

### Participants

After this qualitative study was approved by The Royal Melbourne Hospital Office for Research in August 2022 (QA2022087), potential participants were invited to the study via phone and provided an information sheet via email detailing what their participation would involve. As this project was approved as a Quality Assurance project only verbal consent (rather than written consent) was required from our participants. A convenience sampling method was engaged: potential participants from the haematology ward were invited from a list (provided by a health assistant from the haematology ward) determined by their ability to speak English and their physical and mental capacity to provide informed consent and to participate in an interview, as well as their willingness to participate in the study. Potential participants were phoned during the week post their discharge to invite them to participate in the study and if they agreed to interview and did not become too unwell, they were interviewed in the following week (approximately one – two weeks post-discharge). Approximately one in five of the potential participants dropped out after agreeing to interview because they became too unwell to participate in an interview. Of our 10 recruited participants their age ranges included: 18–20 years old (n = 1); 21–30 years old (n = 2); 31–40 years old (n = 3); 41–50 years old (n = 1); 51–60 years old (n = 2); 61–70 years old (n = 1). Six of our participants were female, and four were male. Nine participants had leukaemia; one participant had a sickle cell condition. Participants’ highest education levels included: secondary school (n = 1); TAFE certification in progress (n = 1), Bachelor degree (n = 6); postgraduate qualification (n = 1); Masters degree in progress (n = 1). Participants were asked of their general comfort level using technology and whether they were comfortable using technology; not comfortable using technology; very comfortable using technology. Participants stated they were very comfortable (n = 8) or comfortable (n = 2) using technology. All but two of our participants were already using the outpatient portal. The two participants who were not using the outpatient portal prior to the study were signed-up to this during the study so that we could assess their experience of outpatient notes once they were discharged from hospital. This study was conducted between January 2023 and January 2024.

### Interviews and analysis

Participants were interviewed for 30–60 min via the Zoom video-conferencing platform. Author SS conducted the interviews remotely to ensure the interviews were conducted in a quiet, private environment and the participant conducted the interviews in the privacy of their homes also. A semi-structured interview guide focussed on the information affordances of the (in)patient portal was employed (see Appendix 1). Interviews were recorded, transcribed and reflexively thematically analysed. This study employs the epistemological lens of contextualism because it concerns knowledge generated from participant experience from a particular context. SS conducted semantic coding in NVivo, describing the dataset and staying close to the participant voice. The dataset was largely inductively analysed – themes were generated from the codes with no *a priori* conceptual framework, though a degree of deduction occurred when framing the dataset in relation to each informational affordance, and in relation to the research questions. SS’ analytic approach is aligned to Braun and Clarke's understanding of reflexive thematic analysis as the generation of knowledge via participant and researcher collaboration.^10,11^ Because of the study's experiential focus, SS engaged a *hermeneutics of empathy* in her interviewing and analytic approach, where the intention was to *understand* participant experience. SS’ PhD and Masters by research in the humanities which drew from qualitative analysis of interviews and her role as a qualitative digital (mental) health researcher and interest in digital ethics informed the analysis. It is significant that SS is not a clinician as this then potentially releases the patient participant from the hierarchical relation with the clinician. However, the position of researcher may be equally problematic in this respect. Authors AB (nursing graduate and director of EMR), BB (nursing graduate, senior clinical informatics officer) and TF (graduate of medicine, chief medical information officer) reviewed the thematic analysis, and their insights informed by their disciplinary backgrounds, roles and interest in increasing transparency in the patient–clinician relationship through patient portals, contributed to its development and finalisation.

## Results

This study resulted in six themes united by an overarching theme that forms part of the title of this paper: *Enabling awareness, agency and participation*. Five of the six themes generated by this analysis capture participant experience of a specific information affordance, its benefit, and, if relevant, how it could be improved. Themes 1, 3, 4 and 5 pertain to participant experience of the individual information affordances of the inpatient portal. Theme 2 focused on access to notes, includes participant experience of both the inpatient and outpatient portal to (as noted above) glean a more comprehensive understanding of participant experience of notes given the restricted access to notes in the inpatient context. Theme 6 broadly captures participant experiences of the inpatient portal via the tablet and recommendations for how it could be further developed. The thematic titles are not participant quotes but are in first-person to emphasise the person-centred point of view. The following themes were generated in this analysis:

1. Results help me understand what is happening, communicate with my clinicians and make informed decisions; immediate access would be beneficial.
2. Notes help me clarify my consultations, engage in my care and know when a clinician has misunderstood me; plain language should replace jargon.
3. Vital signs help me manage my care.
4. Medication information lets me know what I am taking; this can be more meaningful.
5. The schedule enables me to plan for upcoming medical events.
6. The tablet is a user-friendly extension of the hospital environment affording me awareness, agency and participation in my care; it can be developed further.

1. Results help me understand what is happening, communicate with my clinicians and make informed decisions; immediate access would be beneficial.

The ability to access their blood results via the tablet was greatly appreciated by our participants. Haematology patients are informed daily of their results from clinicians who write them on a whiteboard in their room and patients monitor their results to determine how they are responding to their treatment and whether they can be discharged. However, our participants explained that they must wait for or ask the clinician to give them their results, whereas on the tablet they could access them without depending on their clinician. Participants also explained that the results they receive on the whiteboard are not as comprehensive and do not display changes over time as those accessible via the tablet. Participants were particularly interested in the ability to see how their results changed over time, as enabled by the tablet. Those participants who also had access to the inpatient portal via their smartphone, preferred access to their results via the tablet because of this feature that enabled them to see their results mapped over time. Several participants expressed a preference for more immediate access to their results; one participant explained timely access to these is critical when their health is severely compromised.

Participants expressed the importance of access to their results in terms of understanding ‘what's going on’. For some, this understanding impacted them practically where it informed them what they could or could not do:

P2: One of those tests is testing my immune system … it went down … to a level where it was dangerous for me. So, it was very important that I kept track … When you’ve got almost no immune system … you’re not allowed to go outside. You’re not allowed to do bunch of things. So, to know the next morning that it went up … let me know that I could now eat some things and that I was allowed back out of my room.

Access to this information enabled the participant to perform basic actions such as moving or eating without having to wait for a clinician to tell them they can do these things and, in this sense gave them a level of agency.

Participants explained that understanding what is happening in their care positively impacts their wellbeing. P5 expressed the significance of this understanding in terms of ‘having some control’. They expanded,

When … you’re at the mercy of needles and monitors … transfusions and all the rest of it … knowledge is power … It certainly helps my … feeling of wellbeing if I can … have a grasp on what's going on.

P1 conveyed how access to this information helps them in their recovery, gives them a sense of perspective and the agency to arrive at their *own understanding* of what is happening.

P1: It's really important to have an understanding of what's going on … It helps you in your treatment and recovery and to understand why you feel how you feel during your treatment … It's just like – okay, I feel crap because I have a third of what an adult is meant to have … It's just nice to have access to that because … your doctors are treating you based on those type of things … It's nice to … come up with your own sort of understanding … and then understand why your doctors are suggesting something.

This perspective conveys how even if a patient is doing poorly, their awareness of their condition provided by their results brings them relief. It also coveys the importance of the patient understanding their doctor's decisions and feeling part of this decision-making process.

Some participants explained that access to their results via the inpatient portal enabled them to reflect upon this information prior to meeting with their clinician to then engage this information in their meeting.

P5: If you have access to the information before you see your clinician … then you have the ability to think about it and … ask the relevant questions rather than … they just write it on the board, they’re there for five minutes and they say, “Do you have any questions?”. Quite often, you say, “Oh, no, I don’t have any questions,” because you haven’t had time to think about it.

P9 explained that access to this information gave them the confidence to communicate more effectively with their clinician:

We had a more in-depth conversation than I normally would … I could refer to something … That was helpful for me because I get embarrassed and I don't want to come out like an idiot … So that was comforting … It just opened up a few more questions and … points of discussion.

The experience above demonstrates that patients are not just concerned about their health, but also about how their clinicians perceive them and their ability to communicate with their clinicians, all of which impacts their wellbeing.

A participant explained how access to their results helped them collaborate with their clinician to together arrive at an understanding of what they had.

P2: My blood results initiated a lot of discussions because I could see that I was trending down when I’m not on chemo anymore. So, it kind of worried me and then I needed an explanation from my doctor … We talked about it and then we realised, “Oh my God, it's like the last time.” So, *it sort of made us understand what it was that I had*.

This example demonstrates the value for both the patient and clinician of being able to see the results over time, as enabled by the tablet.

Access to their results stimulated further inquiry into the meaning of participants’ results. Several participants explained how they would consider how their lifestyle might impact their results.

P3: When my haemoglobin is low, I can kind of tell what's going on … if I’m not well, or if I’m about to get an infection … I like to pay attention to that … *And now I pay more attention to it because I have access to that information* … You can just compare how it is from the last time … and see if you’ve made lifestyle changes, has it helped? … Is taking these medications helping you? Is it making any difference?

P7: My haemoglobin levels, my platelet levels, liver functions … they’ve been up and down during the periods of recovery … I look at the point in time where maybe they were … really positive results and I start to think about, “Okay, what was I doing at that point? Are there any … environmental factors that might’ve been producing those positive results …?” And conversely, when there's periods … when maybe the results were not as positive … “What was I doing at that point in time? Was I sick? Was I unhealthy? Was I exercising?” … I’m trying to work out … what are the positives and … negatives that could be contributing to the result, and how do I then capitalise on trying to build that into my future lifestyle, food, diet, exercise … work balance, sleep, all that.

The reflection and understanding enabled by access to their results supported these participants in making informed choices in support of their health. Significantly, P3 conveys how access to this information enables them to pay greater attention to their health.

2. Notes help me clarify my consultations, engage in my care and know when a clinician has misunderstood me; plain language should replace jargon.

Participants did not have any recollection of their admission or discharge notes. However, they considered their consultation notes accessed via the outpatient portal a critical resource. They explained that they could refer to these notes for clarification regarding information conveyed to them that they did not process in, or retain from, the consultation, and to be aware of anything that they may have forgotten to raise with their clinician. Participants explained that consultations are stressful, and the notes enable them to reflect upon what was discussed and to engage with their treatment process in their own time. Interestingly some participants also reported that their notes enable them to discern where a clinician has misunderstood them, and, if necessary, to be able to address this misunderstanding. Some participants reported they did not understand the medical jargon included in the notes and that this jargon should be replaced with plain language.

Participants explained that they value access to their notes to review information that they didn’t process in or retain from their consultations. For example,

P7: I love it … *I go and look at them all the time, because sometimes I’m not good at consuming information immediately in that appointment* because … I’m trying to think about … the questions that I … had going into this … My active listening may not be as good as what it normally is so it's nice to … get that summary of information … around what … the outcome of that meeting was.

P4 expressed that though it can be ‘confronting’, they appreciate the option to access their notes to ‘consolidate’ what was discussed in the consultation:

Every time I have a review with the doctor, I’d go back and read the notes … to gauge what we spoke about in that meeting … *It can be* … *confronting to read but I do like having it there* … *to have the option to read what's going on* … to … consolidate what we’ve spoken about … and maybe pick up on a few things that maybe I forgot to mention to the doctor that I could mention for the next time.

Participants explained that because the consultations are often emotionally charged it can be difficult to retain information and therefore access to their notes provides them the ability to refer to this information. Significantly, P6 considered that access to their notes enables them to be ‘engaged with clinical treatment decision-making':

*It's helpful for me to be engaged with the clinical treatment decision-making* … If this treatment doesn’t work, what's next and … *how they’re going to save my life*. And what's the risks or chances of an outcome that is favourable to my long-term health … They’re very emotional consults … High stress environments aren’t usually wonderful to have a retention, so to be able to refer back to that stuff … it's very helpful for me.

This participant conveys the criticality of the notes capturing what was discussed in the consultation so that they can be involved in their care, for, as they express, their life is at stake.

Some participants found mistakes/misunderstandings in their notes. One participant stated that they did not address this mistake with their clinicians as they did not consider it necessary for their care. Another participant remarked they often found mistakes when reading their notes. In one instance they raised a misunderstanding with their clinician, and this action changed the course of their treatment. This experience conveys how sharing this information with patients may not only benefit their wellbeing but may positively impact their treatment.

P3: One of the notes said … I’m very fearful of the red cell exchange, but that's not what I was feeling at the time and that's not what I said to the doctor ... I was then able to clarify … “It's not that I’m scared to do red cell. I’ve done it before. I just don’t feel comfortable having the port. So, any other way of getting the cell exchange is fine.” And that's when … you saw the things hanging out of my neck. So, they put that in … It is good having the notes there because then you can really clarify the situation … For example, I saw Doctor … for the first time and she was reading some of the notes back to me, and I was like, “No, that's not true. That's not really correct. That's not what I said. That's not how I meant it to be.” … *The notes are there* … *for both parties to have a better understanding*, *and it does make sense* … *for me to have a better understanding of what they really think* … that's why I love this app.

Significantly P3 considers that the function of the notes is for both patient and clinician understanding.

3. Vital signs help me manage my care.

Participants explained that it was valuable to access their vital signs via the tablet as the nurse doesn’t always tell them these, or they don’t always process this information at the time it is communicated to them. Participants valued knowing their vital signs whether that be because the information informed their actions in support of their care, or they just appreciated having this level of awareness regarding their health. In both cases – awareness or action – participants conveyed how keeping track of their vital signs enables them to manage their care. This management could take the form of a practical action such as inquiring whether they needed a certain medication based on their blood pressure, or could be a form of emotional self-regulation, as evidenced by the experiences detailed below.

Some participants expressed that access to their vital signs reassured them and gave them a sense of perspective:

P2: All my observations, they don’t normally tend to tell me … But in the app, I can actually see what was the last that was taken. And that was really great too because … we were trying to keep track of my temperature, making sure I wasn’t getting a fever … It was very reassuring for my partner. So, when he came to see me, he would take the iPad, look – “Okay, 36.8, good.” It reassured him and reassured me.

P7: Because I was feeling quite unwell … it also gave me a bit of comfort that I’d go, “You know what? My blood pressure is pretty good and my oxygen is pretty good”. So these markers gave me some comfort … there's still some good elements...the body is still okay.

P6 wanted to keep track of their blood pressure for their diabetes management and referred to this information in their discussion with their clinicians:

They’re keeping an eye on it. I trust them to make good, sound clinical decisions … but it was just so I could have that discussion with them. “Look, my BP has gone back up, what can we do? Can you give me something different rather than the blocker that I was on?”

Here we see how access to their vital signs enabled P6 to actively participate in their care in terms of suggesting treatment.

4. Medication information lets me know what I am taking; this could be more meaningful.

Participants appreciated information on their medications to keep track of what they are taking. As one participant explained, they forget the names of their medications. P3 expressed how access to medication information enabled them to be aware that they were not taking all the medications they needed to and was able to raise this with their clinicians:

I realised they didn’t actually give me my regular medications that I’m supposed to be on based on my pregnancy. They just gave me medications based on my sickle cell … I was able to tell them, “Hey, I’m on amoxicillin. I’m on this. I’m on that.” And then they were able to give me those medications as well.

Several participants considered that this information could be more informative by being clearer, current and including what the medication is for and its side effects:

P8: Is the medication for nausea … for pain … I just see the name, but I don't know what it's used for.

P6: It looked like it was just a cascade of everything I was having … I couldn’t make sense of what they were … I just want to … understand what they were giving me and why they’re giving it to me.

P10: If you could click on them and it went to a link … and listed all their side effects … that’d be great.

5. The schedule enables me to plan for upcoming medical events.

The schedule was valuable to participants because it enabled them to plan for any upcoming medical events such as having scans, rather than being surprised by and unprepared for these events. One participant explains,

P2: There was one morning that … the scan was meant to be at 7:30. So, I knew that I had to wake up around 7:00 … But if I didn’t know, then I would’ve still been sleeping … Or just for my partner to come see me or like friends and family, if I know that I’ve got two scans in the afternoon, then I’ll let them know. I’ll say, “Well, there's no use coming to see me. I have scans all afternoon. Come in the morning.”

P1 explained this function gave them a sense of agency in their care and of being part of the planning process, rather than just feeling that things are being constantly done to them:

I think it's important to be involved in your treatment and to have the understanding of what's going on and it just gives you a sense of … independence … *If things are just happening for you all the time *…* you lose your independence completely*. It's nice to have some sort of schedule … to just know what your day is going to look like … You’re getting so many tests … and sometimes you’re just a bit over it, so a bit of forewarnings would be nice.

One participant explained how they used this feature to know when they could to do their breast pumping for their baby. Another participant explained it is reassuring to have a record of their future medical events, otherwise if they are just told that they will have a scan, they are uncertain that this will in fact happen which causes anxiety.

No participants entered personal events into the schedule because most did not consider this option useful. However, P7 reflected that this could be an impactful affordance:

It's a nice way to be able *to control an environment* that you probably don’t want to be in. I know points in time when I was in the hospital, my partner would come up. You just wait for that dreaded knock on the door. Maybe you’re having a cuddle … because obviously difficult times, then all of a sudden, the door sort of busted in.

Here we see how this affordance could provide a patient a level of control in an environment that is largely outside of their control.

6. The tablet is a user-friendly extension of the hospital environment affording me awareness, agency and participation in my care; it can be developed further.

This theme builds on the previous themes, reiterating their key insights in relation the tablet more generally whilst introducing novel perspectives on the value and impact of accessing the inpatient portal via the tablet. It echoes a key idea offered by one participant that the tablet can be conceived as an extension of the hospital environment. As expressed in various ways by the preceding themes, this theme speaks to how the tablet enables the patient awareness, agency and participation in this environment. All participants agreed that the tablet was easy to use, and several participants expressed how much they ‘loved it’. All participants stated that if they returned to hospital, they would use the tablet again. As with the previous themes, aside from conveying what is beneficial about the tablet, this theme also explores how the inpatient portal via the tablet can be further developed.

Participants compared their experience of the inpatient portal via the tablet to their experience of the inpatient portal via their smartphone. Most of our participants preferred accessing the inpatient portal via the tablet because this device enabled them to see their results and vital signs mapped over time. Another reason why some of the participants preferred the tablet was because it was easier to see the information displayed on a larger screen. Some participants noted that accessing the inpatient portal via the tablet supported patients with poor eyesight.

P6: My preference … would be to use the tablet because it's bigger … The iPhone screen is not ideal for some of this stuff … I’ve got bad eyes … I wear glasses … It's just easier to read on an iPad.

P5: Very easy … The icons are easy to understand … the colour schemes are good and … that's coming from a visual artist's point of view … The size is a good size too for somebody who might wear glasses or … who has vision impairment … I worked in the disability sector … disability accessibility, etc., is really always to the fore with me, and so the icons are large enough and they’re clear enough, simple enough.

However, P5 shared ways the tablet display could be more accessible ‘from a disability point of view’, suggesting ‘black on white’ is ‘better for people with vision impairment’ and having the ability to increase the font size would also be beneficial.

The main reason why participants valued accessing the inpatient portal via the tablet was because it enabled them to view their results over time.

P7: What I really like about the tablet was that … you actually started to have some trend information … so you could actually start to see what was happening … that trend analysis which I really love.

P2 explaining why they would prefer to use the tablet rather than their phone stated:

There was a bit more information … for the extra information that I had access to, those little images where you can see how it's trending.

When asked whether they would use the tablet again P10 stated, ‘Yeah definitely … mainly for the test result graphing. That's definitely the main thing for me’. As detailed in Theme 1, the ability to see their results over time enabled participants to inquire into how their lifestyle may be affecting their results and this in turn enabled them to make more informed choices in support of their health. Most compelling was that for P2 the information captured over time enabled them to collaborate with their clinician to together arrive at understanding of P2's condition.

As explored in the previous themes, the tablet's information affordances supported patient wellbeing through enabling awareness, agency and participation in their care.

P2: I actually loved it … Me and my partner when I’m hospitalised, we’re normally very, very stressed … Having that iPad with all my results, all my last observations and whatnot played a very big part on calming us down, reassuring us, and having answers that we wouldn’t normally have if we didn’t have the iPad.

P7: All of these pieces of information support you becoming more actively involved in your treatment journey. And the more you can become involved, the more you can be educated on what's happening, *the *more you can understand how you can improve outcomes … It means that I’m an active person involved in the process. I’m not just sitting here … *I’m part of the process of recovery* and for me, that's important … versus just leaving it up to chance … Because if you leave it up to chance … that's out of your control. If you’re actively part of it … you have some control in it, *you feel a bit more empowered in the process, and more positive that there's going to be a better outcome*.

Significantly P7 conveys that access to their healthcare information that enables them to be ‘part of the process of recovery’ which makes them optimistic about their recovery.

One participant who considered the tablet an extension of the hospital environment, expressed how this device could function as a form of orientation for a newly admitted patient, informing them of how to negotiate the ward, for example notifying them of areas for eating and entertainment, and any other important information regarding the ward, as well as including key information regarding their condition, treatment and care team. Another participant considered how if the tablet had an emergency button this could assist someone who due to their physically incapacitation could not reach the emergency button on the wall. Another participant stated that the only change they would make to the tablet is having a feature that could enable patients to message their nurses, a perspective that was also shared by nurse and patient participants in Stage 1 of our research. Another participant stated that they would appreciate a function that enabled them to request further discussion regarding their results, so that the patient and the clinician could have a record of this request in case the patient forgets to ask during the ward round. These ideas demonstrate how patient experience could inform portal design.

## Discussion

The information affordances of an (in)patient portal enabled our participants to have greater awareness and therefore understanding of, and by extension greater agency to participate in and manage, their healthcare, all of which supported their wellbeing. To echo one participant, in the hospital environment a patient is constantly *at the mercy* of things being done to them. Patients are acted upon. Indeed, the etymology of the word ‘patient’ ties it to ‘passivity’; as first proposed by Aristotle, *patiency* is the opposite of *agency*.^12^ The definitions of *patiency* and *agency* are ‘the quality or state of being patient or passive’^13^ and ‘the capacity, condition or state of acting or of exerting power’.^14^ To have some sense of control in an environment where most things are outside of their control, to be an active part of the healthcare process, as our participants expressed, contributes to their wellbeing. This perspective is aligned with the more general perspective that self-determination is critical to wellbeing.

A study exploring nurse's perspectives on self-determination for ICU patients, defines self-determination as the ability, ‘to have influence … on your surroundings, to take responsibility for yourself, to be given room to have influence on those decisions that concern you’.^15^ This ICU study also states that patient self-determination involves being seen and heard as an individual, a perspective central to person-centred care. More generally, self-determination theory within psychology locates self-determination as involving autonomy, competence and relatedness all of which contribute to a person's wellbeing.^16^ To translate these key conditions of self-determination in the context of a patient, we can understand autonomy as a patient's level of agency in their healthcare, competence as a patient's level of understanding of their care and relatedness as the patient's ability to communicate with and relate to their care providers. Following this understanding of patient self-determination, the experiences reported in this study demonstrate that the qualities of autonomy, competence and relatedness were supported by the (in)patient portal and contributed to our participants’ wellbeing.

Aside from supporting wellbeing, some experiences detailed in this study indicate that patient participation in their care could improve the quality of the care, and potentially lead to positive health outcomes. Participants reported that they were able to reflect upon their results and make informed choices in support of their health. A participant was able to reflect on their results together with their clinician and collaboratively arrive at an understanding of their condition. Through access to their vital signs, a participant was able to query their clinicians as to whether they needed a certain medication. A participant was able to inform their clinician that they needed to take medications that were not included on their medications list. Through accessing their notes, a participant was able to clarify where the clinician had misunderstood them, and this clarification positively influenced their treatment. These experiences exemplify how when a patient receives information on their healthcare this enables them to reflect on this information and potentially contribute to the knowledge generation in their care and influence their treatment. Significantly the ability to self-reflect is noted as a key factor of *agency*.^17^ The information affordances enabled our participants to reflect on their condition in ways that could support their health and so were agents in this respect.

The experiences detailed above demonstrate how the information affordances of the (in)patient portal can support patient–clinician partnership, which can improve the quality of care. Previous studies have reported that patient engagement with their healthcare information can improve the quality of care for example, in the case of reporting medication errors^3,18^ or reporting mistakes found in notes.^19^ Interestingly, one study reports that through involving them in their care, inpatient portals enable patients to become *better patients*.^20^ This perspective relates to the idea mentioned above regarding *patient competence*. Building on this perspective, we proffer that patient involvement in their care and patient partnership with clinicians supported by the portal, provides clinicians the opportunity to become *better clinicians* – to improve their relationship with patients through increased transparency and to improve the quality of care provided.

*Enablement* is a key term in this paper. In this context it means that via an (in)patient portal the patient has the *choice* to be aware of their healthcare information and the *option* to act on this information, whether this means choosing to do or not to do something in support of their health or being able to collaborate with their clinician in their healthcare. Of course, as one participant expressed, this information can be confronting. Although this was not the case for our participants, sometimes a patient may not want to know, engage or act, as noted elsewhere.^21,22^ The value of an (in)patient portal is that it *gives the patient the option* to access information on and become more involved in, their healthcare.

Our participants were very much interested in seeing their results displayed over time as afforded by the tablet. They explained that this affordance enabled them to inquire into their condition, generate knowledge in relation to it and conceive of how best to manage it. It is worth underlining that this information is visually displayed via graphs which indicates that patients benefit from the visualisation of their healthcare information. It has been previously noted that visualisation supports patient comprehension of complex information.^23^

Another significant affordance of the portal, this time in the outpatient space, was access to consultation notes. These notes were deemed significant to participants because they had a chance to reflect on what had occurred in their consultation and to consider information that they may have missed because of the stressful nature of the meeting. The significance of this information affordance in the outpatient space strongly suggests patients could value access to inpatient notes, such as ward round notes, an idea raised by a doctor participant in Stage 1 of our research.^24^ This participant explained that she conveys the information captured in a ward round note through conversation with patients, but considered patients unable to quickly process this detailed information and that patient access to ward round notes would serve patients in this respect. Patient participants in our Stage 1 research when asked would they want access to their progress notes stated they would want this. Patient desire for inpatient notes is also reported elsewhere.^8^ This study connected the inpatient and outpatient portal due to restricted inpatient access to notes. It points to the need for a more seamless integration of information affordances – specifically in relation to notes – between the inpatient and outpatient portal context, especially for patients who have critical conditions such as our participants.

A report from a study exploring inpatient access to their entire record stated:

Participants navigated to the “clinical notes” feature most frequently, and spent more time using this feature than any other. One participant observed: “The notes were where I was really able to find out what was going on, where all the information was put together … I love being up to speed with [my physician]. When she comes in, she doesn’t have to explain what's going on, because I already know.”^25^

Participants from this study included four patients with heart failure, four post-heart transplant patients and one post-kidney transplant patient. Interestingly, despite this study which demonstrates the benefits of sharing inpatient notes (as is reported in more detail in another paper focused on access to inpatient notes^26^) and the establishment of the effectiveness of the OpenNotes initiative on patient satisfaction and participation in the outpatient space,^27,28^ to our knowledge there are no studies on inpatient portals that include this information affordance. This is perhaps a reflection of clinician resistance to sharing this information with inpatients, as noted in Stage 1 of our research. To repeat the compelling reflection of a participant of this study:

*The notes are *…* essential* … *for both parties to have a better understanding*, *and it does make sense* … *for me to have a better understanding of what they really think*. It would be good for us to have access to the notes.

This perspective presents a massive departure from the clinician understanding reported in Stage 1 of our research of how the notes function primarily as tool of communication between clinicians. It points to the need in the Australian context for clinicians to consider the value the notes hold for patients, who desire to be on the same page as their clinicians for both their psychological wellbeing and physical recovery. To echo the words of a clinician participant from our Stage 1 research, this will require a shift in thinking, for it is not part of Australian hospital culture to share inpatient notes with patients.

This study demonstrates that patients have many ideas as to how the portal's information affordances can be developed including more immediate access to results, plain language replacing jargon in notes, more clear and meaningful information on medications (including what they are for and their side-effects). Our participants considered the benefit of additional affordances via the tablet including ward orientation information, black font on white background, an option for larger font, nurse–patient messaging, a request to discuss results and an emergency button. Patient preference for immediate access to results via a portal has been confirmed by a study where of 8139 participants 96% reported a preference for immediate access.^29^ Patient preference for immediate results as well as plain language replacing jargon in inpatient portal design is also reported elsewhere.^8^ A systematic review states that increased patient input into portal design will improve portal adoption, and echoes patient recommendations reported in our study including more immediate access to results and more meaningful information on medications.^9^

Clinicians should take seriously the perspectives of patients in the design of portals. Typically, portal design has a ‘top-down’ approach where clinicians are the ‘primary stakeholders’ in this design.^30^ As Deering and Baur state,

Optimally, portals should reflect the needs and interests of patients as well as providers, but many portals currently reflect constraints of legacy electronic health record systems and professional perspectives of what patients need.^18^

Although there were patient representatives in discussions regarding what information affordances to include in the RMH inpatient portal prior to its implementation, clinicians were the *primary stakeholders* in these discussions; they overwhelmingly outnumbered the patient representatives. This study that focuses on the significance of patient experience addresses this imbalance. If patients and clinicians are equally represented in consultations for its design, an (in)patient portal has the potential to function as a tool ‘for collaboration and person-centred care’.^18^

(In)patient portals’ information affordances may enable the patient and clinician to come closer being on the same page so that, to echo one participant, *both parties have a better understanding* of a patient's healthcare. *It makes sense* for clinicians and patients to work together – clinicians are experts in their field, and patients, particularly those with critical conditions, as was the case for our participants, are deeply invested in their health. The power of technological mediation is that it has the potential to transform the clinician–patient relationship such that is no longer characterised by an information asymmetry where the clinician knows and the patient does not – a form of epistemological injustice,^31,32^ to one where both parties, under the clinician's expert guidance, are respected as critical participants in healthcare knowledge generation, management and recovery.

## Limitations and future directions

This study concentrated on one area of the hospital; different areas may yield different results. Our participants were comfortable using technology and the majority were well educated. It will be important to understand if patients with low digital literacy and education levels consider the (in)patient portal beneficial. Due to limited access to the ward and the necessity of depending upon people external to the project for recruitment we only recruited 10 patient participants. Although data saturation is a contested concept in RTA,^33^ our project would have benefited from five more participants to generate a more conceptually rich dataset. Despite this limitation we hold that the results generated from the interviews provide insight into how the information affordances of an (in)patient portal benefit patients and how they can be improved. Clinician experiences of the (in)patient portal regarding the three related aspects of its (1) impact on their relationship with patients; (2) ability to support patient-clinician collaboration and (3) ability to improve the quality of care, will be important to explore in future research. Future research on inpatient portals should also explore patient and clinician experience of patient access to inpatient notes, such as ward round notes, due to the dearth of exploration in this area.

## Conclusion

An (in)patient portal's information affordances enable patient awareness, agency and participation in their care, which in turn supports their wellbeing, and in some cases improves the quality of their care and potentially results in positive health outcomes. Participants emphasised that the information affordances gave them some sense of control in an environment that was largely outside of their control. As Australian (in)patient portal implementation is still in its infancy, Australian clinicians will need to harness new attitudes to information sharing and knowledge generation where the patient is not just acted upon but has greater equity when it comes to information access and participation in knowledge generation and the management of their care. Recommendations for how the information affordances could be improved reported in this study should inform future implementations of the (in)patient portal throughout RMH and other healthcare contexts.

## Supplemental Material

sj-docx-1-dhj-10.1177_20552076241288371 - Supplemental material for Enabling awareness, agency and participation: Haematology patient experiences of an (in)patient portal's information affordances Supplemental material, sj-docx-1-dhj-10.1177_20552076241288371 for Enabling awareness, agency and participation: Haematology patient experiences of an (in)patient portal's information affordances by Simone Schmidt, Adam Boulton, Benita Butler and Timothy Fazio in DIGITAL HEALTH

sj-docx-2-dhj-10.1177_20552076241288371 - Supplemental material for Enabling awareness, agency and participation: Haematology patient experiences of an (in)patient portal's information affordances Supplemental material, sj-docx-2-dhj-10.1177_20552076241288371 for Enabling awareness, agency and participation: Haematology patient experiences of an (in)patient portal's information affordances by Simone Schmidt, Adam Boulton, Benita Butler and Timothy Fazio in DIGITAL HEALTH
