# Supplementary material for: Enabling awareness, agency and participation: Haematology patient experiences of an (in)patient portal's information affordances
Source: Digit Health. 2024 Oct 18;10:20552076241288371. doi: 10.1177/20552076241288371 (PMC11526392; doi:10.1177/20552076241288371)
Supplement: sj-docx-1-dhj-10.1177_20552076241288371 - Supplemental material for Enabling awareness, agency and participation: Haematology patient experiences of an (in)patient portal's information affordances [file sj-docx-1-dhj-10.1177_20552076241288371.docx]

MyChart Bedside Interview Guide

[Prior to recording]

The study will be briefly explicated. Participants will be reminded that the interviews will be recorded, and they will be asked to verbally consent to this. Questions focus on the distinct information affordances. Follow up questions will be asked to fully understand the participant’s experience.

[Recording]

1. What is your age range (e.g. 31-40)?
2. What is your highest level of education?
3. How would you describe your level of comfort using technology? - comfortable, very comfortable, not comfortable.
4. What was your experience of using MyChart Bedside via the tablet?
5. *Vital signs.* What was your experience of having access to your vital signs (e.g. blood pressure, heart-rate, temperature) throughout your hospital stay?
6. *Results.* What was your experience of having access to your test results (e.g. blood tests) throughout your hospital stay?
7. *Notes.* What was your experience of accessing your medical notes in hospital (admission notes) and once discharged (discharge and consultation notes)?
8. *Medications.* What was your experience of having information on your medications throughout your hospital stay?
9. *Schedule.* What was your experience of seeing medical events in your schedule during your hospital stay? Did you add any personal events to this schedule?
10. If you were in hospital again, would you use MyChart Bedside via the tablet again?
11. Is there anything else you would like to tell me about your experience of MyChart Bedside during your hospital stay?
12. Do you have any comments/questions in relation to this study?
